# Community-centred eco-bio-social approach to control dengue vectors: an intervention study from Myanmar

**DOI:** 10.1179/2047773212Y.0000000057

**Published:** 2012-12

**Authors:** Khin Thet Wai, Pe Than Htun, Tin Oo, Hla Myint, Zaw Lin, Axel Kroeger, Johannes Sommerfeld, Max Petzold

**Affiliations:** 1 Ministry of Health, Myanmar; 2 WHO/TDR, Geneva, Switzerland; 3 Sahlgrenska Academy, University of Gothenburg, Sweden

**Keywords:** Partnership-driven ecosystem management intervention, Dengue vector breeding, Informed decision-making, Integrated vector management, Community empowerment

## Abstract

**Objectives:**

To build up and analyse the feasibility, process, and effectiveness of a partnership-driven ecosystem management intervention in reducing dengue vector breeding and constructing sustainable partnerships among multiple stakeholders.

**Methods:**

A community-based intervention study was conducted from May 2009 to January 2010 in Yangon city. Six high-risk and six low-risk clusters were randomized and allocated as intervention and routine service areas, respectively. For each cluster, 100 households were covered. Bi-monthly entomological evaluations (i.e. larval and pupal surveys) and household acceptability surveys at the end of 6-month intervention period were conducted, supplemented by qualitative evaluations.

**Intervention description:**

The strategies included eco-friendly multi-stakeholder partner groups (*Thingaha*) and ward-based volunteers, informed decision-making of householders, followed by integrated vector management approach.

**Findings:**

Pupae per person index (PPI) decreased at the last evaluation by 5.7% (0.35–0.33) in high-risk clusters. But in low-risk clusters, PPI remarkably decreased by 63.6% (0.33–0.12). In routine service area, PPI also decreased due to availability of Temephos after Cyclone Nargis. As for total number of pupae in all containers, when compared to evaluation 1, there was a reduction of 18.6% in evaluation 2 and 44.1% in evaluation 3 in intervention area. However, in routine service area, more reduction was observed. All intervention tools were found as acceptable, being feasible to implement by multi-stakeholder partner groups.

**Conclusions:**

The efficacy of community-controlled partnership-driven interventions was found to be superior to the vertical approach in terms of sustainability and community empowerment.

## Introduction

In Myanmar, dengue has been occurring since the 1970s with epidemics reported in 3–4 years cycles.^1^ The vector-borne disease control programme from the Department of Health, Myanmar is responsible for establishing effective disease and vector surveillance systems, to undertake disease prevention through selective, stratified, and integrated vector control and to increase community awareness and collaboration. Pupal surveys able to target the most productive container types^2^ are not included in their routine activities and other novel approaches of integrated vector management^3^ are not recognized. Continuous community efforts for integrated vector control together with environmental management can be expensive and without the support of key stakeholders, sustainability is questionable.^4^,^5^ A strategy of targeted source reduction was proposed^2^ directed solely against highly productive containers that may be sufficient to control dengue vectors; the cost-effectiveness of such an approach was confirmed later in a multi-country study.^6^ Present vertical programmes are inefficient and educational messages are often unclear; staffing levels, capacity building, management and organization, funding, and community engagement tend to be insufficient.^7^ Thus, the approach towards enhancing community involvement^8^ is important although the effectiveness of such strategies is variable.^9^ During the baseline studies of eco-bio-social research programme to control dengue vector breeding^5^ in Yangon city, the most productive water container types for *Aedes* pupae as a proxy measure for vector density in households were identified (see section on ‘Results’) and could be specifically targeted for vector control interventions. It was decided to conduct an intervention study with the objectives to build up and analyse the feasibility, process, and effectiveness of a partnership-driven ecosystem management intervention in reducing vector densities and constructing sustainable partnerships among multiple stakeholders.

## Methods

### Timeline and study site

A community-based intervention study using a cluster randomization balanced design was carried out from May 2009 to January 2010 in Yangon city (96°09′ longitude and 16°48′ latitude). The total population as of 2008–2009 in the city was around 6.8 million and the population density was 666 persons per square kilometre. Two peri-urban eco-settings in Yangon Region were selected purposively and stratified on the basis of reported dengue incidence for 3 years: North Dagon (zone 1 in the north-eastern part) with low to moderate dengue transmission and Insein (zone 2 in the north-western part) with moderate to high dengue transmission.

### Research methods

#### Weather variables

Temperature, rainfall, and relative humidity throughout the period of the intervention were collected by the Kabar Aye weather station in Yangon, approximately 10 km away from zone 1 and 8 km away from zone 2. This station was under the Department of Meteorology and Hydrology, NayPyiTaw.

#### Sampling of study neighbourhoods

In the baseline survey in phase 1, 20 urban neighbourhoods or ‘clusters’ were randomly selected and analysed.^5^ However, in phase 2, the cluster background information was used to stratify six high-risk clusters and six low-risk clusters out of 20 clusters. These selected clusters were randomly allocated to intervention and routine service areas, respectively. To have a balanced design in each area, three high-risk and three low-risk clusters were selected at random. Each cluster included roughly, 100 households. Considering the flight range of mosquitoes, a buffer zone of at least 100 m around the intervention and routine service areas was taken into account.

#### Sample size

For the post-intervention cross-sectional analysis of intervention clusters versus routine service clusters, the following sample sizes were needed for a significance level of 5% and a power of 80%. As for pupae per person index (PPI), by anticipating a mean level of 0.3 in routine service clusters and 0.1 in intervention clusters with a standard deviation of 0.1, the needed number of cluster per arm was 4–6.

#### Qualitative research

For focus group discussions (FGDs) of householders and dengue volunteer groups, 10 and 6, respectively were organized in intervention clusters to underscore satisfaction with and opinions about the intervention. Furthermore, 10 in-depth interviews were conducted with eco-health friendly groups (EFGs), Health Department and Municipal services on feasibility and sustainability of the intervention programme. Additionally, one multi-stakeholder discussion with 20 participants from different social sectors focussing the importance and sustainability issues was conducted in the intervention neighbourhoods. All FGDs and in-depth interviews were audio-taped after a written informed consent. The analysis was facilitated by using SAS^2^ methods^10^ for ‘priority options’ and ‘feasibility’. Themes and sub-themes were analysed manually and triangulated with quantitative data.

#### Household surveys and statistical analysis

A formal household survey for the acceptance of intervention tools was done in six intervention clusters at the end of phase 2 by using pre-tested interview forms. Altogether 555 householders were interviewed. The safety of the intervention was measured by recording side effects by volunteers. Data entry was carried out by using EPI DATA version 3.0. SPSS version 17.0 was used for the analysis following range and consistency checks. The unit of analysis is clusters.

### Intervention methods

#### Rationale for the intervention package

Phase 1 findings include varying causes of dengue vector breeding, most productive container types, current vector control measures, limited mobilization of health and health-related networks, weaknesses in supervision of volunteers, and their capacity pointed out to the need of changing the strategy and developing a new vector control model for effective source reduction at low cost either chemical alone or in combination with non-chemical and biological measures as an integrated approach. It was also felt that the vertical control strategy should be replaced by a community-based model for larval control operations leaded by a multi-stakeholder partner group with the Township Health Department (THD) being the focal point. The community-based model covered interested stakeholder partner groups and volunteers together with highly motivated householders to augment the routine vector control activities by the Township Health Department.

#### Selection and capacity building of partners

1. EFGs (*Thingaha*) were formed, one in each selected intervention cluster by recruitment of existing viable groups led by ward authorities jointly with midwives, members of Maternal and Child Welfare Association, and credible and trusted persons and school teachers. Each group comprised one leader and four core members. They were trained for information dissemination and how to manage vector control tools. Their functions included to set local targets for household coverage, to liaise between Township Dengue Control Committee (TDCC) and householders, and to organize/mobilize householders to accept interventions and environmental management, supervision of volunteers, and deployment of intervention tools. *Thingaha* groups distributed pictorial booklets and pamphlets regarding dengue already in use by Maternal and Child Welfare Association members.
2. Local manufacturers were explained to produce tightly fitted lid covers at low cost which should serve as a model to be replicated by community members.
3. Ward-based volunteers were selected by EFG, 10 in each cluster to participate actively in controlling dengue vectors. Each volunteer was responsible for local target setting of households to be visited, to provide information and to assist in the inspection and removal of larvae. Each volunteer was provided with a record book to note the vector control tool preference of each household and any problems if encountered in each visit. Their activities were monitored weekly by EFG. Two days training on communication of dengue issues and the use of cotton-net sweepers and a half day of field demonstration was done by the research team jointly with TDCC.
4. An intensive awareness-raising for the local communities was done through group discussions. Interpersonal communication reinforced by information leaflets and booklets distributed in house to house visits was carried out explaining three sets of intervention tools for targeted containers (chemical or non-chemical measures or both). The TDCC scheduled household monitoring visits weekly either on Wednesday or Saturday accompanied by EFG. Monthly meetings and feedback regarding constraints and solutions were carried out in the THD throughout the intervention for three times.

#### Vector control tools

Selective vector control measures were applied in intervention clusters for 6 months. Among 10 different types of containers, four produced more than 80% of all pupae (‘productive containers’) as a proxy for adult mosquitoes: drum (200 l), cement tank (100–950 l), ceramic jar (50–100 l), and spiritual bowl (1–2 l). Four intervention tools were applied according to the type of container and peoples’ preferences: pyriproxyfen sand granules and *Bti* as chemical control, lid covers and cotton-net sweepers as mechanical control, dragon fly nymphs as biological control (container variety of dragon fly nymphs, *B. geminator* (Rambur) was seeded into cement tanks), and waste-collection bags for removing discarded small containers. The list of options for each container type was shown to heads of the household to choose which one to use in their home, and then to sign a form which hung on the entrance of the premises so that the entomological team could apply the desired intervention. The research team used an information matrix to explain the risk and benefits of each intervention tool so as to make choices according to their preferences. In routine service clusters, usual control measures were carried out. As mentioned before, these were basically larviciding with temephos (Abate) very much enhanced by the response to the cyclone disaster. This activity was done in every cluster prior to phase 2.

#### Entomological surveys and the intended measurement of effectiveness

The effectiveness of integrated vector management that covered the use of combined chemical and non-chemical methods and environmental management through social mobilization efforts was measured by comparison of pupa and larva indices between intervention and routine service clusters during three rounds of evaluation surveys. The PPI (total number of *Ae. aegypti* pupae by number of inhabitants) was used as a proxy indicator for adult density. The pupae per container index was used as an indicator for the infestation levels of different container types. As secondary measures, the larval indices were used: Breteau index, house index, and container index. Assuming the sero-prevalence rate as 33%, the estimated threshold level of PPI of 0.19 at the average temperature of 30°C at the given period was kept in mind.^11^ Before the start of the intervention study, cyclone Nargis struck the city in May 2008 so that all research activities had to be postponed.The public response included massive larviciding of all water containers by the control programme. For saving resources, the intervention areas were covered by the project staff and the control clusters by personnel from Ministry of Health using temephos treatment of water containers which was donated by foreign aid for the relief operations. This situation limited the possibilities of determining the effectiveness of our intervention package against purely untreated control group.

#### Performance measurement

The quality of the delivery of the intervention package was assessed by observing programme performance and analysing whether targets were reached. Furthermore, the adequate programme performance in terms of timeliness, rational use of resources, competencies, relations between volunteers and householders, level of community satisfaction, and coordination between volunteers and eco-health friendly partner groups was assessed in a qualitative way.

### Ethical considerations

The study was approved by the Technical and Ethical Review Committee, Department of Medical Research (Lower Myanmar) and from the Ethical Review Committee, WHO, Geneva. The informed consent was obtained from householders and multi-stakeholder partner groups during data collection.

## Results

### Characteristics of the study population

The majority of householders from the six intervention clusters participated in structured interviews (555/600, 92.5%). The total number of dwellers was 3054 with an average of 5.5 individuals per household. Only 10.3% of household heads were unemployed and 15–24% were children under 15 years. Most of the inhabitants were Buddhists except in one cluster in which equal proportions of Buddhists and Christians were found. Those characteristics confirmed what had been found in the whole study population in phase 1.^5^

### Productive containers to be targeted by the intervention

The most productive water container types for *Aedes* pupae at baseline were man-made spiritual worshipped bowls, cement tanks, and flower vases. In the subsequent surveys, metal or cement drums replaced the flower vases (Table 1). The most infested container types by *Aedes* larvae were rarely the most productive ones for adult mosquitoes. Total number of pupae in all containers reduced to 18.6% in evaluation 2 and 44.1% in evaluation 3 in intervention area. However, in routine service area, more reduction was observed. Variations in type of productive containers in both areas were limited which means almost similar. At baseline, less than 20% of water containers were properly covered in intervention clusters and less than 5% in clusters of the routine service area (Table 1). Householders’ confidence and trust in community groups improved and their misperceptions and negative attitudes towards dengue vector control activities disappeared or were rarely mentioned in group discussions after interventions.

**Table 1:** Vector breeding places and productivity in clusters at baseline and three rounds of evaluation

Characteristic		Intervention area (I) (*n* = 6)			Routine service area (R) (*n* = 6)		
	Baseline (wet)*	Eval 1 (qet)	Eval 2 (semi-wet)	Eval 3 (dry)	Eval 1 (wet)	Eval 2 (semi-wet)	Eval 3 (dry)
Number of households	2000	593	592	592	600	599	600
Total number of water containers	18 510	4802	4857	4460	5110	5135	4697
% of indoor containers	37.5	60.3	61.4	60.9	61.9	63.6	64.7
% of tap water filled	81.6	91.5	95.4	99.3	82.3	96.2	98.6
% of containers fully covered	14.5	16.5	16.0	15.7	4.4	3.8	3.4
Most frequent container types (% of all container types)	Flower vase (48.7%)	Flower vase (47.9%)	Flower vase (50.5%)	Flower vase (49.8%)	Flower vase (51.8%)	Flower vase (53.8%)	Flower vase (56.8%)
	Cement tank† (14.3%)	Cement tank (16.5%)	Cement tank (16.0%)	Cement tank (16.8%)	Cement tank (14.2%)	Cement tank (14.2%)	Cement tank (14.7%)
	Drum/barrel (12.4%)	Drum/barrel (14.6%)	Drum/barrel (14.0%)	Drum/barrel (14.6%)	Drum/barrel (11.3%)	Drum/barrel (10.9%)	Drum/barrel (10.6%)
Total number of pupae in all containers	2155	295	240	165	1117	874	493
% of reduction in pupae	—	—	18.64	44.06	—	21.75	55.86
Most productive container types (% of all pupae)	Spiritual bowl (51.7%)	Drum/barrel (34.1%)	Drum/barrel (38.2%)	Cement tank (31.0%)	Cement tank (38.9%)	Drum/barrel (41.4%)	Cement tank (41.0%)
	Cement tank† (19.5)	Ceramic jar (19.0%)	Flower vase (19.7%)	Bucket (26.8%)	Ceramic jar (26.1%)	Cement tank (16.8%)	Drum/barrel (27.6%)
	Flower vases (7.2%)	Cement tank (16.3%)	Ceramic jar (17.2%)	Drum/barrel (24.7%)	Drum/barrel (17.2%)	Ceramic jar (16.4%)	Spiritual bowl (21.9%)

**Notes:** *Source: Arunachalam *et al.*, 2010, p. 179.

†Cement tanks included cement drums in baseline studies.

### Preferred choices and strategies of interventions

At baseline, there was little collaboration and partnership among stakeholders in dengue vector control and the community was a passive recipient of public health interventions. The intervention package mainly delivered by EFG improved the understanding and shared responsibility among local authorities and the community. Distributing pamphlets and booklets and assisting people in the application of targeted container interventions strengthened the leadership of EFG and the development of sense of ownership by community members. Managerial skills, leadership roles, and the participation of EFG and volunteers in activities to reduce dengue vectors were strengthened. A practical ‘matrix-based decision-making tool’ illustrating vector control options to facilitate the decision by heads of household was well accepted. According to Table 2, combined measures were most frequently favoured (44.8% of cluster dwellers), while chemical measures were the second choice (34.2% of cluster dwellers) and mechanical measures the third choice (16.5% of cluster dwellers). Biological measures were preferred in a combined package but rarely alone.

**Table 2:** Initial choice of intervention measures in six clusters in relation to type of water containers

Initial choice expressed by cluster dwellers (*n* = 6)	Average	Percent
Combined (chemical, mechanical, and biological measures)	45	44.8
Chemical measures (pyriproxyfen and *Bti*) only	34	34.2
Mechanical measure (lid covers and cotton-net sweepers) only	17	16.5
Biological measure (dragon fly nymphs) only	1	0.7
Refusal	4	3.8

### Stakeholder processes

Six multi-stakeholder groups involved in dengue prevention and control activities existed at baseline in form of ‘Dengue Control Committees’ in the Townships of Yangon Region (TDCC) since 2003–2004. Dengue control in a collective way involving actively local communities was meant to be carried out at that time but was felt to be inadequate. Therefore, in the first phase of this research programme, power, legitimacy, interests, and interactions towards controlling dengue vectors in each stakeholder group were thoroughly discussed and analysed. In phase 2, the importance as well as favourable and unfavourable conditions related to the six strategic options to reduce dengue vector breeding was discussed and scores were given to ascertain the feasibility and sustainability of each option. These strategic options included: formation of EFG, recruitment of ward-based volunteers, informed choices of vector control tools for most productive containers, targeted container approach for implementation of chemical measures, inspection and removal of larvae and pupae, keeping separate waste collection bags for water retaining discarded materials, and integrated use of mechanical and biological measures. The preferred options were: (1) to train volunteers; (2) to target and manage productive containers; and (3) to use waste collection bags. Discussants realized also the importance of forming multi-stakeholder groups at ward level which was feasible and necessary for extended ownership of the programme.

### Intervention effect on people’s knowledge, attitudes, and practices

At baseline, the overall knowledge of 2000 respondents on dengue-related issues was high^5^ but their container management practices were inadequate especially for productive large size containers. Qualitative evaluations after the intervention captured that people’s awareness of appropriate vector control options for specific containers was highly improved as well as positive attitudes towards joint actions. At the end of the intervention period (see Table 3), nearly 45% of cluster dwellers accepted pyriproxyfen alone or in combination with other measures. They perceived the chemical as being extremely beneficial and nearly 60% had full confidence in it. Of cluster dwellers using *Bti* for their ceramic bowls, only 28% perceived it to be extremely beneficial. Lid covers were accepted by 52 households per cluster and 60% of cluster dwellers were fully confident to use them continuously which was important for vector control in the area. Dragon fly nymphs were found in 12 households per cluster but nearly 60% of cluster dwellers found those nymphs as being extremely beneficial and perceived them as being important in removal of larvae and pupae from their water containers. Nearly 42% of cluster dwellers perceived waste collection bags as extremely beneficial for them and 52% was fully confident for continuity in use. There were no differences between high- and low-risk clusters. The results indicated that people were less enthusiastic about *Bti* and cotton-net sweepers.

**Table 3:** Acceptability of six intervention tools in intervention clusters (*n* = 6)

Cluster dwellers who accepted*	Average of clusters	% of acceptance
Pyriproxyfen		
Very desirable/extremely beneficial	31	44.6
Definitely feasible in households	31	43.9
Very important	28	39.4
Confident	42	59.3
*Bti*		
Very desirable/extremely beneficial	6	28.0
Definitely feasible in households	6	32.5
Very important	6	28.2
Confident	10	49.2
Lid covers		
Very desirable/extremely beneficial	27	51.6
Definitely feasible in households	26	50.5
Very important	24	46.1
Confident	31	60.5
Cotton-net sweepers		
Very desirable/extremely beneficial	15	32.7
Definitely feasible in households	14	31.2
Very important	14	30.3
Confident	14	30.3
Dragon fly nymphs		
Very desirable/extremely beneficial	7	57.4
Definitely feasible in households	7	59.4
Very important	7	58.0
Confident	8	64.3
Waste collection bags		
Very desirable/extremely beneficial	31	41.9
Definitely feasible in households	29	38.1
Very important	31	41.5
Confident	39	53.0

**Note:** *Multiple responses; totals do not add up to 100.

In the FGDs and observations following the intervention, it became clear that householders’ responsibility in managing dengue vector breeding sites was enhanced. They became interested in the inspection and removal of larvae in their homes; they used lid covers and cotton-net sweepers and scrubbed the containers and changed the water regularly in contrast to responses at baseline when household members did not regularly scrub and change water especially of the large containers. It became also clear that cultural barriers persisted in the management of spiritual bowls and that, against original expectations, the use of dragon fly nymphs for cement tanks still needs promotion.

### Reduction in dengue vector density

**Figure 1:**
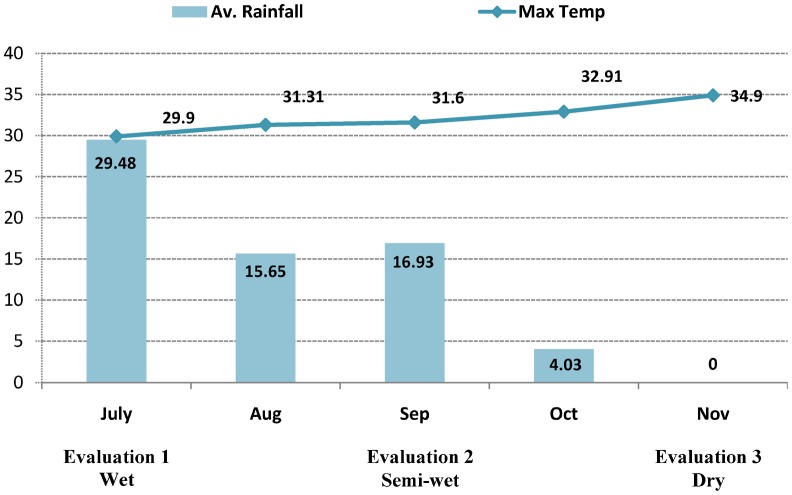
Distribution of average monthly rainfall, maximum day time temperature, and three rounds of entomological evaluation.

**Figure 2:**
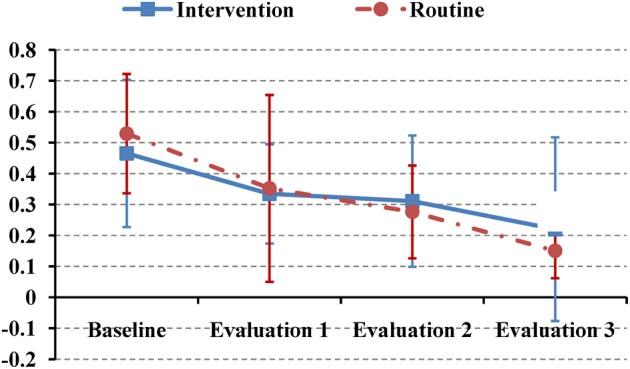
Mean pupae per person index (PPI) and 95% confidence intervals at baseline and in three rounds of evaluation surveys in clusters of the community-centred intervention (*n* = 6) and the areas with routine larviciding (*n* = 6).

After the entomological baseline survey during the wet season of 2007 in phase 1, three entomological evaluations were conducted, the first during the wet season, the second during the semi-wet season, and the third during the dry season (Fig. 1). As mentioned before, after the baseline survey, cyclone Nargis struck the city and as a response by the vector control services a massive larviciding programme with temephos (Abate) was launched. Our community-centred multi-stakeholder intervention programme was as good in reducing vector densities (using as the PPI as the main outcome measure) as the massive intervention with temephos in the routine service areas (Fig. 2). The PPI decreased from 0.34 at the first evaluation to 0.23 at the last evaluation (32% reduction) in intervention clusters and from 0.33 to 0.15 (54.5% reduction) in routine service clusters. The PPI decreased in the last evaluation by 5.7% (0.35–0.33) in high-risk clusters but much more in low-risk clusters (63.6% reduction; 0.33–0.12). Similarly, the Stegomyia indices decreased: container index from 27.7 to 19.4, Breteau index from 49.7 to 27.9, and house index from 6.3 to 3.7. The same level of reduction was detected in the clusters where routine larviciding activities with Temephos were carried out especially during 2008 (the year of cyclone Nargis) and continued in 2009.

## Discussion

### Multi-stakeholder partnerships in urban eco-health interventions and sustainability

It became clear during our study period that purely vertical interventions in dengue vector control are not sustainable particularly if they are built upon ‘crisis management’ as in our case the response to the dengue threat after cyclone Nargis. This involved massive external emergency relief funds that included five metric tons of larvicides (temephos 1% sand granules) initially.^12^ Therefore, continued supply of temephos was available through the year 2009, which coincided with our intervention year, and still in 2010 when the stocks were emptied. Over 10 years prior to 2008, the routine services could not include temephos for larviciding due to limited resources and they will probably not be able to do it in the near future. Community participation in dengue vector control is desirable as it has prospects of better programme sustainability even if external funds are not anymore available. Equally important in our study was the multi-partnership approach: the EFG acted as the liaison between the community and the THD. They established close relationships among different partners and helped to build and maintain the sense of ownership. The eco-health component was particularly evident in the recognition of the vector ecology in particular in the identification and management of productive breeding places.^5^ This led to the development of a matrix illustrating vector control options according to productive water container types and was used for strengthening household decision-making on the specific intervention they preferred. It took only 1–2 days for volunteers to identify in 600 households, people’s preference of vector control options. Community acceptance was high throughout the 6 months of intervention for targeted containers. The disposition of providing active contributions, time, and space for removal of larvae/or pupae was enhanced and not solely depended upon basic health staff who had time constraints and excessive work load. Targeting productive container types was as effective in reducing the PPI as targeting all types in routine service areas but had lower implementation costs.^6^

Active involvement of the community in project design and implementation influenced the sustainability of health interventions in Cuba.^13^ Sustainability of the current intervention package depends upon: (1) political commitment and continuing support by the local governance; (2) the extent of interest of the community, acceptance, and their active participation including the development of a sense of responsibility in using appropriate mechanical control tools (such as lid covers and cotton-net sweepers); and (3) additional programme costs. These conditions were largely fulfilled in our study area. The leadership of EFGs was successful as they achieved that ward authorities developed a strong commitment in problem identification at baseline and in scheduling, motivating people, and distributing intervention materials and later on in monitoring the implementation and results. However, the attrition of EFG members and volunteers required not only continuous motivation through good leadership by the THD but also a system of replacement.

Local municipal authorities assisted in ward-based waste collection but challenges were sometimes inadequate manpower and vehicles. The recycling of water retaining discarded materials required private sector involvement. For maintaining community interest in managing mechanical control tools, the continuing support by EFGs for households was essential particularly by working with local manufacturers for any demand to replace or to purchase new water container lids and other tools at affordable costs. Continuing use of pyriproxyfen (approximately 50 USD/kg) will mean additional programme costs but these can be obtained through the coordination between Regional Health Department and City Development Committee for the purchase of pyriproxyfen at lower cost by subsidization compared to temephos which was actually cheaper that is approximately 30 USD/kg. Moreover, the high acceptability of pyriproxiphen by users due to lack of smell may facilitate the programme demand and sustainability.

The PPI decreased higher in low-risk compared to high-risk dengue areas. In high-risk clusters, the distance between each household and also to adjacent control clusters was closer than in low-risk clusters according to cluster background information at baseline,^5^ so that *Aedes* mosquitoes in high-risk areas could fly from control clusters into intervention clusters (‘spill over effect’ which increases the vector density in intervention clusters); this was much less the case in low-transmission clusters.

### Other challenges in controlling dengue virus transmission

The PPI in three rounds of entomological surveys was reduced but not below the theoretical threshold limits for epidemic transmission of 0.19 (Fig. 2),^14^ indicating a persistent transmission risk. The exact correlation was unknown but the increase in vectors in the wet season coincided with the increase in dengue cases (dengue season). Another challenge is the need for high coverage and repeated larviciding as shown in northern Argentina.^15^ Mass larviciding was however successful in Cambodia.^16^ Water storage practices, still existing despite improvement in municipal water supply, and population growth are other factors favouring degue spread. Demographic growth was noted in the study areas during 5 years (2005–2009). The population increase was 9.7% in the six intervention areas but only 1.5% in the non-intervention clusters. Thus, complex public health response is imperative for effective reduction in the long run as noted in this project by incorporating integrated vector management principles^3^ rather than focussing on larviciding only as in vertical programmes.

## Conclusion

The efficacy of the community and multi-partnership-based intervention was equivalent to the massive vertical larviciding organized as a vertical programme in the aftermath of cyclone Nargis. However, in terms of sustainability and empowerment of communities and other stakeholders, the partnership approach with targeted container interventions was found to be superior to the vertical approach. The policy implications are: partnerships between community and municipal services are to be strengthened further in terms of waste segregation, adequate and continuous water supply, and improved water management. Vector control efforts are required to focus on the most productive water container types and environmental sanitation activities dealing with solid waste disposal focussed at integrated vector management. Further research is required on establishing the long-term sustainability of the intervention package and its delivery in high-risk transmission areas.
